# Hybrid Beamforming Design for Self-Interference Cancellation in Full-Duplex Millimeter-Wave MIMO Systems with Dynamic Subarrays

**DOI:** 10.3390/e24111687

**Published:** 2022-11-18

**Authors:** Gengshan Wang, Zhijia Yang, Tierui Gong

**Affiliations:** 1 State Key Laboratory of Robotics, Shenyang Institute of Automation, Chinese Academy of Sciences, Shenyang 110016, China; 2 Key Laboratory of Networked Control Systems, Chinese Academy of Sciences, Shenyang 110016, China; 3 Institutes for Robotics and Intelligent Manufacturing, Chinese Academy of Sciences, Shenyang 110169, China; 4 University of Chinese Academy of Sciences, Beijing 100049, China; 5 Engineering Product Development Pillar, Singapore University of Technology and Design, Singapore 487372, Singapore

**Keywords:** millimeter wave, full-duplex, hybrid beamforming, self-interference cancellation, dynamic subarrays

## Abstract

Full-duplex (FD) millimeter-wave (mmWave) multiple-input multiple-output (MIMO) communication is a promising solution for the extremely high-throughput requirements in future cellular systems. The hybrid beamforming structure is preferable for its low hardware complexity and low power consumption with acceptable performance. In this paper, we introduce the hardware efficient dynamic subarrays to the FD mmWave MIMO systems and propose an effective hybrid beamforming design to cancel the self-interference (SI) in the considered system. First, assuming no SI, we obtain the optimal fully digital beamformers and combiners via the singular value decomposition of the uplink and downlink channels and the water-filling power allocation. Then, based on the obtained fully digital solutions, we get the dynamic analog solutions and digital solutions using the Kuhn–Munkres algorithm-aided dynamic hybrid beamforming design. Finally, we resort to the null space projection method to cancel the SI by projecting the obtained digital beamformer at the base station onto the null space of the equivalent SI channel. We further analyze the computational complexity of the proposed method. Numerical results demonstrate the superiority of the FD mmWave MIMO systems with the dynamic subarrays using the proposed method compared to the systems with the fixed subarrays and the half-duplex mmWave communications. When the number of RF chains is 6 and the signal-to-noise ratio is 10 dB, the proposed design outperforms the FD mmWave MIMO systems with fixed subarrays and the half-duplex mmWave communications, respectively, by 22.4% and 47.9% in spectral efficiency and 19.9% and 101% in energy efficiency.

## 1. Introduction

Millimeter-wave (mmWave) communication, using the abundantly available spectral resources from 30 to 300 GHz, is one of the key candidate technologies for future cellular systems [1,2,3,4]. Although the path loss at mmWave frequency is severe, the short wavelength enables more antennas to be packed in a small physical dimension. Thus, massive multiple-input multiple-output (MIMO) technology can be leveraged to achieve large-scale multiplexing and highly directional beamforming gains. To further improve the spectral efficiency (SE), the full-duplex (FD) wireless communication, which has the potential to double SE by allowing transmitting and receiving signals simultaneously in the same frequency band [5], has been integrated with mmWave communications [6,7]. The FD mmWave MIMO systems can sufficiently utilize the spectrum, space, and time-frequency resources to support extremely high data rates [7,8,9].

Due to the high cost and power consumptions of mmWave radio frequency (RF) chains, the conventional fully digital beamforming that requires a dedicated RF chain connected to each antenna is infeasible for mmWave massive MIMO systems [1]. The analog beamforming, connecting all antennas via phase shifters to a single RF chain, is the simplest beamforming scheme to reduce the hardware constraints. However, this scheme only supports single-user and single-stream communication, leading to low SE performance [1,10]. Provided the compromise between system performance and hardware complexity, hybrid analog-digital beamforming has been proposed. In the hybrid beamforming scheme, the analog beamforming is high-dimensional and implemented by phase shifter networks, making the number of required RF chains fewer than that of antennas, and thus, this scheme has low cost and power consumption; the digital beamforming is low-dimensional and completed at the baseband, allowing the hybrid beamforming to support multi-user and multi-stream communications. According to the connections between RF chains and antennas, hybrid beamforming structures can be classified into the fully connected structures (FCSs) and partially connected structures (PCSs). The FCS can achieve full beamforming gains, for each antenna is connected to all RF chains via phase shifters. Thus, this structure has been widely adopted and well investigated [1,11,12,13,14]. Although the FCS is more hardware-efficient than the fully digital beamforming, this structure also requires a large number of phase shifters, resulting in relatively high hardware complexity and power consumption. To further reduce the hardware complexity, the PCS, in which each RF chain is connected to a non-overlapping subset of antennas, has been proposed and studied [12,15,16,17,18,19,20,21]. Furthermore, based on different partitions of the antenna array, the PCS can be categorized into fixed subarrays (FSs) and dynamic subarrays (DSs). In the FS, the antenna subset is fixed, sacrificing large beamforming gains [12,15,16,17]. In the DS, the antenna array can be partitioned into subarrays associated with different RF chains dynamically and adaptively, improving the SE performance compared to the FS [17,18,19,20].

When transmitting while receiving signals simultaneously in the same frequency band, the transmitted signals of the FD node will leak to its own receiver, termed self-interference (SI), and overwhelm the desired signals received from far ends [5]. If the SI cannot be effectively canceled to an acceptable level, the residual SI will result in a serious SE loss in the FD system. Therefore, the biggest challenge in achieving FD communications is the SI. The FD systems in the sub-6 GHz bands have been first investigated, and the SI can be canceled by jointly using the passive and active methods in the propagation, analog, and digital domains [5,22,23,24,25]. For FD mmWave systems, the authors in [26] established the first comprehensive FD mmWave platform through the proposed SI cancellation methods at the antenna, analog, and digital frontends, which can cancel the SI by more than 80 dB and improve the SE to outperform the half-duplex (HD) by approximately 70%. This work demonstrates the feasibility of the FD mmWave communications. However, this work only considers the single-input single-output (SISO) system and is difficult to generalize to the FD mmWave MIMO system due to the extremely high hardware and computational complexity. Though the fully digital beamforming to cancel the SI via spatial suppression is a preferable scheme in the sub-6 GHz FD MIMO systems [27,28], the high cost and power consumption of the required large number of RF chains make this scheme impracticable in the FD mmWave MIMO system. To further reduce the hardware constraints, analog beamforming-based FD mmWave MIMO systems have been proposed [6,29,30,31]. However, this scheme only supports single-user and single-stream communication, seriously limiting the performance and application of the FD mmWave MIMO communications.

Therefore, the hybrid beamforming, capable of improving performance while keeping relatively low hardware complexity, is more feasible than fully digital and analog beamforming in the FD mmWave MIMO system. For the bidirectional FD mmWave topology, the authors in [32] proposed a joint hybrid beamformer and combiner design, aiming to cancel the SI while preserving the dimensions of transmitted signals. This proposed design was able to cancel the SI by 30 dB. The work in [33] exploited the alternating projection method to design the hybrid beamforming, which can effectively cancel the SI in the analog domain and significantly improve the SE. The authors in [34] proposed a hybrid beamforming design to cancel the SI in the analog domain and maximize the SE simultaneously. In [35], the authors proposed an angular-based joint precoding and combining method, which can effectively improve the SI cancellation and increase the desired signal power. The work in [36] focused on the FD mmWave systems with finite-resolution phase shifters and the dynamic range of analog-to-digital converters (ADCs). The authors proposed different hybrid beamforming and combining methods, which can cancel the SI sufficiently to avoid the saturation of ADCs at the receiver and achieve good SE performance. For the FD mmWave base station (BS) topology, the authors in [37] proposed a hybrid beamforming cancellation method, significantly enhancing the SE. The authors further extended this proposed beamforming cancellation method to the scenario that considers the limited dynamic ranges of ADCs in [38] and the wideband scenario in [39]. The work in [40] considered the limited dynamic ranges of a low-noise amplifier (LNA) per antenna and ADCs per RF chain and presented an effective hybrid beamforming design to avoid receiver-side saturation and achieve high SE. In [41,42], considering the FD mmWave MIMO system with low-resolution phase shifters, the authors resorted to the penalty dual decomposition (PDD) framework and block coordinate descent (BCD) method to obtain a nearly optimal hybrid beamforming design. For the FD mmWave relay topology, the authors in [43] proposed a hybrid beamforming design to cancel the SI. The work in [44] presented an alternating method with close-form solutions to design robust hybrid transceivers under imperfect channel estimations. However, all the above works only consider the FD mmWave MIMO systems with FCSs that require large numbers of phase shifters. To evaluate the performance of FD mmWave MIMO systems with FSs, a SI cancellation-based hybrid beamforming method was proposed in [45], which can achieve better SE than the HD but worse SE than the FD system with FCSs. The authors in [46] leveraged the PDD framework to design the robust hybrid analog-digital transceivers for the FD mmWave multi-cell system with FSs in the presence of channel state information (CSI) error. However, this work assumed that the SI could be sufficiently suppressed via the existing SI cancellation methods in [5,24,25]. Although these above works have proposed some effective hybrid beamforming methods for the FD mmWave MIMO systems with classical FCSs or FSs, the DSs, having been proven to have benefits on both SE and energy efficiency (EE) in the HD mmWave communications [17,18,19,20], have not been considered and evaluated in the FD mmWave systems.

In light of the above related works on the FD mmWave communications, the main contributions of this paper are summarized as follows:

- We introduce the DSs to the FD mmWave MIMO systems, where the BS and users are equipped with multiple antennas, adopt the DSs, and support multi-stream communications. To the best of our knowledge, the hybrid beamforming design for the considered system has not been well investigated.
- We propose an effective three-step hybrid beamforming design based on SI cancellation for the considered system. In the first step, under the assumption of no SI, we obtain the optimal fully digital solutions using the singular value decomposition (SVD) of the uplink (UL) and downlink (DL) channels and the water-filling power allocation algorithm. In the second step, we leverage the Kuhn–Munkres-assisted dynamic hybrid beamforming design to decompose every obtained fully digital solution into an analog component corresponding to the DS and a digital component. In the third step, we first establish the equivalent SI channel and then project the digital beamformer at BS onto the null space of the equivalent SI channel to null out the SI.
- We analyze the computational complexity of the proposed three-step hybrid beamforming design. Numerical results validate that the FD mmWave MIMO system with DSs using the proposed hybrid beamforming design can achieve better SE and EE compared to the systems with FSs and the HD mmWave system.

*Organizations:*Section 2 introduces the system model and problem formulation. Section 3 proposes the three-step SI cancellation-based hybrid beamforming design and analyzes its computational complexity. Section 4 presents the numerical results to evaluate the SE and EE of the proposed design. Section 5 concludes this paper.

*Notations:* In this paper, the boldface lower-face and upper-face letters denote the column vectors and matrices, respectively. $A^{T}$, $A^{H}$, $A^{-1}$, $\mathrm{Tr}\left(A\right)$, ${∥A∥}_{F}$, and ${∥A∥}_{0}$ represent the transpose, Hermitian transpose, inverse, trace, Frobenius norm, and zero norm of matrix A. $A\left(i,:\right)$, $A\left(:,j\right)$, and $A\left(i,j\right)$, respectively, stand for the *i*-th row, the *j*-th column, and the element on the *i*-th row and *j*-th column of matrix A. $C^{M\times N}$ represents the space of M×N complex matrices. $I_{N}$ is the N×N identity matrix, and $0_{N}\left(0_{M\times N}\right)$ denotes the N×N(M×N) zero matrix. $\mathrm{CN}\left(\mu ,\Sigma \right)$ represents the distribution of the circularly symmetric complex Gaussian random vector variable with mean μ and covariance matrix Σ. $N\left(\mu ,\sigma^{2}\right)$ denotes the real Gaussian random variable with mean μ and variance $\sigma^{2}$. N(A) denotes one set of orthogonal bases for the null space of matrix A. U[a,b] denotes the uniform distribution in [a,b]. The operator |·| represents the absolute value of a scalar or the determinant of a matrix or the number of elements in a set. The operators E{·}, Re{·}, and svd(·) refer to taking the expectation, real part, and singular value decomposition of the argument, respectively.

## 2. System Model and Problem Formulation

### 2.1. System Model

**Figure 1:**
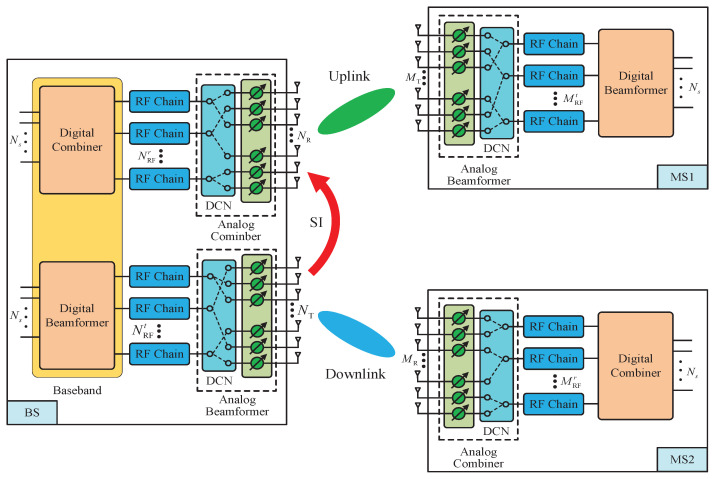
The full-duplex mmWave MIMO systems with dynamic subarrays.

Consider a single-cell narrowband mmWave cellular system, as illustrated in Figure 1, including one BS working in FD mode and two users (i.e., MS1 and MS2) operating in HD mode. The considered system is a classical FD BS topology, and the DSs are applied at the BS and users. The BS is equipped with $N_{T}$ transmit antennas, $N_{R}$ receive antennas, $N_{\mathrm{RF}}^{t}$ transmit RF chains as well as $N_{\mathrm{RF}}^{r}$ receive RF chains. MS1 is equipped with $M_{T}$ transmit antennas and $M_{\mathrm{RF}}^{t}$ transmit RF chains, and MS2 is equipped with $M_{R}$ receive antennas and $M_{\mathrm{RF}}^{r}$ receive RF chains. The number of data streams is $N_{s}$. As the BS is FD capable, it receives signals transmitted from MS1 in the UL and simultaneously transmits signals to MS2 in the DL. Due to the severe path loss of mmWave channels, we assume the distance between MS1 and MS2 is sufficiently far, and thus, the interference between them can be neglected.

At MS1, the transmitted symbol vector $s_{U}\in C^{N_{s}\times 1}$ satisfying $E\left\{s_{U}s_{U}^{H}\right\}=I_{N_{s}}$ is first precoded by the digital beamformer $F_{\mathrm{BBU}}\in C^{M_{\mathrm{RF}}^{t}\times N_{s}}$ at the baseband and then processed by the analog beamformer $F_{\mathrm{RFU}}\in C^{M_{T}\times M_{\mathrm{RF}}^{t}}$ at mmWave frequency implemented using phase shifters. Thus, the final transmitted signal is $x_{U}=F_{\mathrm{RFU}}F_{\mathrm{BBU}}s_{U}$. Similarly, at BS, the transmitted symbol vector $s_{D}\in C^{N_{s}\times 1}$ such that $E\left\{s_{D}s_{D}^{H}\right\}=I_{N_{s}}$ is successively processed by the digital beamformer $F_{\mathrm{BBD}}\in C^{N_{\mathrm{RF}}^{t}\times N_{s}}$ and the analog beamformer $F_{\mathrm{RFD}}\in C^{N_{T}\times N_{\mathrm{RF}}^{t}}$, yielding the final transmitted signal $x_{D}=F_{\mathrm{RFD}}F_{\mathrm{BBD}}s_{D}$.

Due to working in FD mode, the BS receives not only the desired signals from MS1 through the UL channel $H_{U}\in C^{N_{R}\times M_{T}}$ but also its own transmitted signal (i.e., SI) through the SI channel $H_{\mathrm{SI}}\in C^{N_{R}\times N_{T}}$. The received signal is first processed by the analog combiner $W_{\mathrm{RFU}}\in C^{N_{R}\times N_{\mathrm{RF}}^{r}}$ at the mmWave frequency, followed by the digital combiner $W_{\mathrm{BBU}}\in C^{N_{\mathrm{RF}}^{r}\times N_{s}}$ at the baseband. Thus, the final received signal is

(1) $$\begin{array}{cc} {\tilde{y}}_{U}= & \;W_{\mathrm{BBU}}^{H}W_{\mathrm{RFU}}^{H}H_{U}F_{\mathrm{RFU}}F_{\mathrm{BBU}}s_{U}+W_{\mathrm{BBU}}^{H}W_{\mathrm{RFU}}^{H}H_{\mathrm{SI}}F_{\mathrm{RFD}}F_{\mathrm{BBD}}s_{D}+W_{\mathrm{BBU}}^{H}W_{\mathrm{RFU}}^{H}n_{U}\\ = & \;\underset{\mathrm{the}\;\mathrm{disired}\;\mathrm{signal}\;\mathrm{from}\;\mathrm{MS}1}{\underset{︸}{W_{U}^{H}H_{U}F_{U}s_{U}}}+\underset{\mathrm{SI}}{\underset{︸}{W_{U}^{H}H_{\mathrm{SI}}F_{D}s_{D}}}+\underset{\mathrm{noise}}{\underset{︸}{W_{U}^{H}n_{U}}}, \end{array}$$

where $n_{U}∼\mathrm{CN}\left(0_{N_{R}\times 1},\sigma_{U}^{2}I_{N_{R}}\right)$ denotes the additive white Gaussian noise (AWGN) at the BS. $W_{U}=W_{\mathrm{RFU}}W_{\mathrm{BBU}}$ is the overall hybrid combiner at the BS, and $F_{U}=F_{\mathrm{RFU}}F_{\mathrm{BBU}}$ and $F_{D}=F_{\mathrm{RFD}}F_{\mathrm{BBD}}$, respectively, represent the overall hybrid beamformer at MS1 and the BS. Since we assume no inter-user interference, MS2 only receives the desired signal from the BS. After the processing of the analog combiner $W_{\mathrm{RFD}}\in C^{M_{R}\times M_{\mathrm{RF}}^{r}}$ and digital combiner $W_{\mathrm{BBD}}\in C^{M_{\mathrm{RF}}^{r}\times N_{s}}$, the final received signal is given by

(2) $$\begin{array}{cc} {\tilde{y}}_{D}= & \;W_{\mathrm{BBD}}^{H}W_{\mathrm{RFD}}^{H}H_{D}F_{\mathrm{RFD}}F_{\mathrm{BBD}}s_{D}+W_{\mathrm{BBD}}^{H}W_{\mathrm{RFD}}^{H}n_{D}\\ = & \;\underset{\mathrm{the}\;\mathrm{disired}\;\mathrm{signal}\;\mathrm{from}\;\mathrm{BS}}{\underset{︸}{W_{D}^{H}H_{D}F_{D}s_{D}}}+\underset{\mathrm{noise}}{\underset{︸}{W_{D}^{H}n_{D}}}, \end{array}$$

where $H_{D}\in C^{M_{R}\times N_{T}}$ denotes the DL channel from the BS to MS2, and $n_{D}∼\mathrm{CN}\left(0_{M_{R}\times 1},\sigma_{D}^{2}I_{M_{R}}\right)$ is the AWGN at MS2. Furthermore, $W_{D}=W_{\mathrm{RFD}}W_{\mathrm{BBD}}$ represents the overall hybrid combiner at MS2.

### 2.2. Channel Model

For the UL and DL channels, the distance between the BS and MS1 (MS2) satisfies the far-field conditions. Thus, we adopt the extended Saleh–Valenzuela model [47] to characterize the narrowband mmWave channel, which is expressed as

(3) $$\begin{array}{c} H=\sqrt{\frac{N_{t}N_{r}\gamma }{N_{c}N_{l}}}\sum_{c=1}^{N_{c}}\sum_{l=1}^{N_{l}}\alpha_{cl}a_{r}\left(\theta_{cl}^{r}\right)a_{t}{\left(\theta_{cl}^{t}\right)}^{H}, \end{array}$$

where $N_{t}$ and $N_{r}$ denote the number of transmit and receive antennas. $N_{c}$ is the number of scattering clusters, and $N_{l}$ is the number of propagation paths in each cluster. γ denotes the path loss, and $\alpha_{cl}∼\mathrm{CN}\left(0,1\right)$ is the complex gain of the *l*-th path in the *c*-th cluster. $\theta_{cl}^{r}$ and $\theta_{cl}^{t}$ represent the azimuth angle of arrival (AoA) and angle of departure (AoD) of the *l*-th path in the *c*-th cluster, and their corresponding normalized receive and transmit antenna array response vectors are $a_{r}\left(\theta_{cl}^{r}\right)$ and $a_{t}\left(\theta_{cl}^{t}\right)$, respectively. Based on the settings at BS and MSs, we easily obtain the expressions of $H_{U}$ and $H_{D}$ by modifying the number of transmit antennas, receive antennas, clusters and rays, path losses and gains, as well as the AoAs and AoDs.

For the SI channel, as the transmit and receive antenna arrays at BS are relatively close, their distance cannot meet the far-field conditions. Thus, the near-field effect must be taken into consideration. As in [6,32,37], we model the mmWave SI channel as the following Rician fading channel

(4) $$\begin{array}{c} H_{\mathrm{SI}}=\sqrt{\frac{\kappa }{\kappa +1}}H_{\mathrm{SI}}^{\mathrm{los}}+\sqrt{\frac{1}{\kappa +1}}H_{\mathrm{SI}}^{\mathrm{nlos}}, \end{array}$$

where κ is the Rician factor, denoting the ratio of the power in the near-field portion to that in the far-field portion. $H_{\mathrm{SI}}^{\mathrm{los}}$ is the near-field component of the mmWave SI channel, characterized by the spherical-wave propagation model as follows:

(5) $$\begin{array}{c} H_{\mathrm{SI}}^{\mathrm{los}}\left(m,n\right)=\frac{\rho }{r_{mn}}e^{-j\frac{2\pi }{\lambda }r_{mn}}, \end{array}$$

where ρ is a power normalization constant such that $E\{∥H_{\mathrm{SI}}{∥}_{F}^{2}\}=N_{T}N_{R}$, λ is the wavelength, and $r_{mn}$ is the distance between the *m*-th receive antenna and the *n*-th transmit antenna, which can be calculated using the cosine theorem according to Figure 2 in [6]. $H_{\mathrm{SI}}^{\mathrm{nlos}}$ is the far-field component of the SI channel and can also be modeled using (3).

### 2.3. Dynamic Subarrays

In DSs, the analog beamformer or combiner is composed of the dynamic connection network (DCN) and phase shifters, as plotted in Figure 1. The DCN, usually implemented via a switch network, provides dynamic and flexible connections between RF chains and antennas [17,18,19,20]. The DSs require that each antenna is connected to only one RF chain and each RF chain is connected to at least one antenna, introducing the following additional nonconvex constraints on the analog beamformers and combiners:

(6a) $$∥F_{\mathrm{RFU}}{\left(i,:\right)∥}_{0}=1,\;i=1,2,\ldots ,M_{T},$$

(6b) $$∥F_{\mathrm{RFD}}{\left(i,:\right)∥}_{0}=1,\;i=1,2,\ldots ,N_{T},$$

(6c) $$∥W_{\mathrm{RFU}}{\left(i,:\right)∥}_{0}=1,\;i=1,2,\ldots ,N_{R},$$

(6d) $$∥W_{\mathrm{RFD}}{\left(i,:\right)∥}_{0}=1,\;i=1,2,\ldots ,M_{R},$$

(6e) $$∥F_{\mathrm{RFU}}{\left(:,j\right)∥}_{0}\geq 1,\;j=1,2,\ldots ,M_{\mathrm{RF}}^{t},$$

(6f) $$∥F_{\mathrm{RFD}}{\left(:,j\right)∥}_{0}\geq 1,\;j=1,2,\ldots ,N_{\mathrm{RF}}^{t},$$

(6g) $$∥W_{\mathrm{RFU}}{\left(:,j\right)∥}_{0}\geq 1,\;j=1,2,\ldots ,N_{\mathrm{RF}}^{r},$$

(6h) $$∥W_{\mathrm{RFD}}{\left(:,j\right)∥}_{0}\geq 1,\;j=1,2,\ldots ,M_{\mathrm{RF}}^{r}.$$

### 2.4. Problem Formulation

In this paper, we assume the data streams are Gaussian signaling, and thus, the achievable SE of BS and MS2 are, respectively, defined as

(7a) $$\begin{array}{cc} R_{U}= & \log_{2}\left|I_{N_{s}}+C_{U}^{-1}W_{U}^{H}H_{U}F_{U}F_{U}^{H}H_{U}^{H}W_{U}\right|, \end{array}$$

(7b) $$\begin{array}{cc} R_{D}= & \log_{2}\left|I_{N_{s}}+C_{D}^{-1}W_{D}^{H}H_{D}F_{D}F_{D}^{H}H_{D}^{H}W_{D}\right|, \end{array}$$

where $C_{U}=W_{U}^{H}H_{\mathrm{SI}}F_{D}F_{D}^{H}H_{\mathrm{SI}}^{H}W_{U}+\sigma_{U}^{2}W_{U}^{H}W_{U}$ and $C_{D}=\sigma_{D}^{2}W_{D}^{H}W_{D}$.

Under the assumption that CSI is perfectly known at the BS and the ADCs have sufficiently large dynamic ranges, the problem of interest is to jointly design the hybrid beamformers and combiners at BS and MSs to maximize the overall SE of the FD mmWave MIMO systems with DSs, which is formulated as follows:

$$\begin{array}{cc} \max_{A}\;\;\; & R_{U}+R_{D} \end{array}$$

(8a) $$\begin{array}{cc} s.t.\;\;\;\; & W_{\mathrm{BBU}}^{H}W_{\mathrm{RFU}}^{H}H_{\mathrm{SI}}F_{\mathrm{RFD}}F_{\mathrm{BBD}}=0_{N_{s}}, \end{array}$$

(8b) $$\begin{array}{cc} \; & ∥F_{\mathrm{RFU}}F_{\mathrm{BBU}}{∥}_{F}^{2}=P_{U}, \end{array}$$

(8c) $$\begin{array}{cc} \; & ∥F_{\mathrm{RFD}}F_{\mathrm{BBD}}{∥}_{F}^{2}=P_{D}, \end{array}$$

(8d) $$\begin{array}{cc} \; & |F_{\mathrm{RFU}}\left(i,j\right)|=1,\;\forall \left(i,j\right)\in F_{U}, \end{array}$$

(8e) $$\begin{array}{cc} \; & |F_{\mathrm{RFD}}\left(i,j\right)|=1,\;\forall \left(i,j\right)\in F_{D}, \end{array}$$

(8f) $$\begin{array}{cc} \; & |W_{\mathrm{RFU}}\left(i,j\right)|=1,\;\forall \left(i,j\right)\in W_{U}, \end{array}$$

(8g) $$\begin{array}{cc} \; & |W_{\mathrm{RFD}}\left(i,j\right)|=1,\;\forall \left(i,j\right)\in W_{D}, \end{array}$$

$$\begin{array}{cc} \; & \left(6a––h\right) \end{array}$$

where $A=\{F_{\mathrm{RFU}},F_{\mathrm{BBU}},W_{\mathrm{RFU}},W_{\mathrm{BBU}},F_{\mathrm{RFD}},F_{\mathrm{BBD}},W_{\mathrm{RFU}},W_{\mathrm{BBD}}\}$ is the set of optimization variables. Constraint (8a) is the ideal SI cancellation requirement that the SI can be completely suppressed via the hybrid beamforming and combining at the BS. Constraints (8b) and (8c), respectively, denote the transmit power constraint at MS1 and BS, where $P_{U}$ and $P_{D}$ are the corresponding maximum transmit powers. Constraints (8d–e) ((8f–g)) are the unit-modulus constraints on the nonzero elements of the analog beamformers (combiners), where $F_{U}$ and $F_{D}$ ($W_{U}$ and $W_{D}$) are the sets of nonzero elements of the analog beamformers (combiners). Constraints (6a–h) are the constraints introduced by the DSs, among which constraints (6a–d) guarantee that each antenna is only connected to one RF chain and constraints (6e–h) ensure that each RF chain is connected to at least one antenna.

## 3. Proposed SI Cancellation Based Hybrid Beamforming Design

Subject to the ideal SI cancellation constraint, the transmit power constraints, the unit-modulus constraints, and the constraints introduced by the DSs, problem (8) is a nonconvex optimization problem and extremely difficult to solve directly [48]. In this section, we propose a feasible and effective hybrid beamforming design to cancel the SI in the FD mmWave MIMO systems with DSs. The proposed hybrid beamforming design includes the following three steps, which will be introduced in detail.

### 3.1. The First Step—Fully Digital Beamforming When Assuming No SI

Let ${\tilde{F}}_{U}\in C^{N_{T}\times N_{s}}$ and ${\tilde{F}}_{D}\in C^{M_{T}\times N_{s}}$ denote the fully digital beamformer at MS1 and the BS, and ${\tilde{W}}_{U}\in C^{N_{R}\times N_{s}}$ and ${\tilde{W}}_{D}\in C^{M_{R}\times N_{s}}$ be the fully digital combiner at the BS and MS2. As we only consider the fully digital beamforming at this step, the transmit power constraints at MS1 and the BS can be further written as $∥{\tilde{F}}_{U}{∥}_{F}^{2}=P_{U}$ and $∥{\tilde{F}}_{D}{∥}_{F}^{2}=P_{D}$, and the nonconvex constraints (8d–g) and (6a–h) can be removed. When assuming no SI, the constraint (8a) is satisfied, and thus, the first term in $C_{U}$ can be neglected. Consider the SVD of the UL channel as $H_{U}=U_{U}\Sigma_{U}V_{U}^{H}$, where $U_{U}\in C^{N_{R}\times N_{R}}$ and $V_{U}\in C^{M_{T}\times M_{T}}$ are the unitary matrices and $\Sigma_{U}\in C^{N_{R}\times N_{T}}$ is a rectangular diagonal matrix composed of the singular values in decreasing order. Then, according to [49], the optimal fully digital beamformer ${\tilde{F}}_{U}^{*}$ at MS1 corresponds to the first $N_{s}$ columns of $V_{U}$ with water-filling power allocations, and the optimal fully digital combiner ${\tilde{W}}_{U}^{*}$ at BS corresponds to the first $N_{s}$ columns of $U_{U}$, which are expressed as

(9a) $${\tilde{F}}_{U}^{*}=V_{U}\left(:,1:N_{s}\right)T_{U}^{\frac{1}{2}},$$

(9b) $${\tilde{W}}_{U}^{*}=U_{U}\left(:,1:N_{s}\right),$$

where $T_{U}$ is a diagonal matrix whose each diagonal element denotes the allocated power to the corresponding data stream using the water-filling algorithm, satisfying $\mathrm{Tr}\left(T_{U}\right)=P_{U}$. Similarly, if the SVD of the DL channel is $H_{D}=U_{D}\Sigma_{D}V_{D}^{H}$, where $\Sigma_{D}$ is a rectangular diagonal matrix whose main diagonal elements are the singular values in decreasing order, and $U_{D}\in C^{M_{R}\times M_{R}}$ and $V_{U}\in C^{N_{T}\times N_{T}}$ are the unitary matrices, then the optimal fully digital beamformer ${\tilde{F}}_{D}^{*}$ at the BS and the corresponding optimal fully digital combiner ${\tilde{W}}_{D}^{*}$ at MS2 are given by

(10a) $${\tilde{F}}_{D}^{*}=V_{D}\left(:,1:N_{s}\right)T_{D}^{\frac{1}{2}},$$

(10b) $${\tilde{W}}_{D}^{*}=U_{D}\left(:,1:N_{s}\right),$$

where $T_{D}$ is a diagonal power allocation matrix with elements derived by the water-filling algorithm such that $\mathrm{Tr}\left(T_{D}\right)=P_{D}$.

### 3.2. The Second Step—Hybrid Beamforming for the Dynamic Subarrays When Assuming No SI

In point-to-point mmWave MIMO communications, the hybrid beamforming design for maximizing the system SE is approximately solved by minimizing the Euclidean distance between the optimal fully digital and hybrid solutions [11,12,13]. When assuming no SI, the UL and DL communications are parallel and independent, and thus we can leverage the above conclusion to obtain the hybrid beamformers and combiners from the corresponding fully digital solutions obtained in the first step. Without loss of generality, we take the hybrid beamformer design from ${\tilde{F}}_{D}^{*}$ at the BS as an example. Hence, this hybrid beamformer design subproblem is approximately equivalent to the following matrix decomposition problem:

(11a) $$\min_{F_{\mathrm{RFD}},F_{\mathrm{BBD}}}\;\;\;∥{\tilde{F}}_{D}^{*}-F_{\mathrm{RFD}}F_{\mathrm{BBD}}{∥}_{F}^{2}$$

(11b) $$s.t.\;\;\;\;∥F_{\mathrm{RFD}}F_{\mathrm{BBD}}{∥}_{F}^{2}=P_{D},$$

(11c) $$|F_{\mathrm{RFD}}\left(i,j\right)|=1,\;\forall \left(i,j\right)\in F_{D},$$

(11d) $$∥F_{\mathrm{RFD}}{\left(i,:\right)∥}_{0}=1,\;i=1,2,\ldots ,N_{T},$$

(11e) $$∥F_{\mathrm{RFD}}{\left(:,j\right)∥}_{0}\geq 1,\;j=1,2,\ldots ,N_{\mathrm{RF}}^{t}.$$

The above problem is also nonconvex and very complicated. As proved in [12], we can first not consider the transmit power constraint (11b) to solve the problem and then normalize the final digital beamformer to satisfy this constraint, which can simplify this problem. To solve the above hybrid beamformer design problem for the DSs, we resort to the Kuhn–Munkres algorithm-assisted hybrid beamforming design proposed in [21], which iteratively updates the analog and digital solutions as follows.

#### 3.2.1. Analog Beamformer Design

As DSs require that each antenna is only connected to one RF chain, there is only one nonzero element in each row of $F_{\mathrm{RFD}}$. We assume the *i*-th antenna is connected to the $r_{i}$-th RF chain. When fixing the other variables, the subproblem with respect to $F_{\mathrm{RFD}}$ can be further equivalently written as

(12) $$\begin{array}{cc} \min_{B}\;\;\; & \sum_{i=1}^{N_{T}}{∥{\tilde{F}}_{D}^{*}\left(i,:\right)-F_{\mathrm{RFD}}\left(i,r_{i}\right)F_{\mathrm{BBD}}\left(r_{i},:\right)∥}_{F}^{2}\\ s.t.\;\;\; & \left(11c––e\right) \end{array}$$

where $B={\left\{F_{\mathrm{RFD}}\left(i,r_{i}\right),r_{i}\right\}}_{i=1}^{N_{T}}$. Thus, solving the above problem is to determine the connection of each antenna (i.e., $r_{i}$) and the corresponding element in the analog beamformer (i.e., $F_{\mathrm{RFD}}\left(i,r_{i}\right)$). Since there is only one nonzero element with a unit modulus in each row of $F_{\mathrm{RFD}}$, the above problem can be further separated into $N_{T}$ subproblems with respect to each nonzero element. Hence, we can update the only nonzero element in each row of $F_{\mathrm{RFD}}$ independently and separately. With the other variables fixed and the constant terms removed, the subproblem with respect to the *i*-th antenna is given by

(13a) minCTrFBBD(ri,:)FBBD(ri,:)H−2Re{TrF˜D*(i,:)FBBD(ri,:)HFRFD(i,ri)H}

(13b) $$s.t.\;\;\;\;|F_{\mathrm{RFD}}\left(i,j\right)|=1,\;j\in \{1,2,\ldots ,N_{\mathrm{RF}}^{t}\},$$

(13c) $$\;\;\;\;∥F_{\mathrm{RFD}}{\left(i,:\right)∥}_{0}=1,$$

where $C=\{F_{\mathrm{RFD}}\left(i,r_{i}\right),r_{i}\}$. To minimize the objective function (13a), the optimal antenna connection $r_{i}^{*}$ and the unit-modulus nonzero element $F_{\mathrm{RFD}}^{*}\left(i,r_{i}^{*}\right)$ are

(14a) $$\begin{array}{cc} \; & r_{i}^{*}=\min_{r_{i}}\mathrm{Tr}\left(F_{\mathrm{BBD}}\left(r_{i},:\right)F_{\mathrm{BBD}}{\left(r_{i},:\right)}^{H}\right)-2\left|{\tilde{F}}_{D}^{*}\left(i,:\right)F_{\mathrm{BBD}}{\left(r_{i},:\right)}^{H}\right|, \end{array}$$

(14b) $$\begin{array}{cc} \; & F_{\mathrm{RFD}}^{*}\left(i,r_{i}^{*}\right)=\frac{{\tilde{F}}_{D}^{*}\left(i,:\right)F_{\mathrm{BBD}}{\left(r_{i}^{*},:\right)}^{H}}{|{\tilde{F}}_{D}^{*}\left(i,:\right)F_{\mathrm{BBD}}{\left(r_{i}^{*},:\right)}^{H}|}. \end{array}$$

We can use the above methods to determine the optimal connections of each antenna (i.e., ${\left\{r_{i}^{*}\right\}}_{i=1}^{N_{T}}$) and the corresponding analog beamformer $F_{\mathrm{RFD}}^{*}$. As the solutions are obtained without considering constraint (11e), they can guarantee that each antenna is only connected to one RF chain but may not satisfy that each RF chain is connected to at least one antenna. If there are some RF chains connected to no antennas, we should reallocate some antennas to these RF chains. Assuming the number of RF chains connected to no antennas is $N_{\mathrm{RF}}^{t0}$, as in [21], we first establish the antenna reallocation cost matrix and then exploit the extended Kuhn–Munkres algorithm to determine how to select and reallocate $N_{\mathrm{RF}}^{t0}$ antennas to these $N_{\mathrm{RF}}^{t0}$ RF chains. For clarity, we omit the specific antenna reallocation, the details of which can be found in [21].

#### 3.2.2. Digital Beamformer Design

When the other variables are fixed, the subproblem with respect to the digital beamformer $F_{\mathrm{BBD}}$ is

(15) minFBBDTrFBBDHFRFDHFRFDFBBD−2Re{TrFBBDHFRFDHF˜D*}

where the constant terms are not included. The above problem is an unconstrained convex optimization problem with respect to $F_{\mathrm{BBD}}$. Checking the first-order optimality condition yields the optimal solution $F_{\mathrm{BBD}}^{*}$ as

(16) $$\begin{array}{c} F_{\mathrm{BBD}}^{*}={\left(F_{\mathrm{RFD}}^{H}F_{\mathrm{RFD}}\right)}^{-1}F_{\mathrm{RFD}}^{H}{\tilde{F}}_{D}^{*}. \end{array}$$

We update the analog beamformer $F_{\mathrm{RFD}}$ and digital beamformer $F_{\mathrm{BBD}}$ iteratively and alternatively until some termination criterion is satisfied. Then, we normalize the obtained digital beamformer $F_{\mathrm{BBD}}$ as follows:

(17) $$\begin{array}{c} F_{\mathrm{BBD}}=\sqrt{P_{D}}\frac{F_{\mathrm{BBD}}}{∥F_{\mathrm{RFD}}F_{\mathrm{BBD}}{∥}_{F}}, \end{array}$$

which ensures satisfying the transmit power constraint (11b).

Similarly, we can directly use the same method to obtain the hybrid beamformer $F_{\mathrm{RFU}}$ and $F_{\mathrm{BBU}}$ from the fully digital beamformer ${\tilde{F}}_{U}^{*}$. Meanwhile, this method can also be exploited to obtain the hybrid combiners from the fully digital combiners ${\tilde{W}}_{U}^{*}$ and ${\tilde{W}}_{D}^{*}$ obtained in the first step, but without the power normalization.

### 3.3. The Third Step—Null Space Projection to Cancel the SI

As it is usually difficult to satisfy the assumption that there is no SI, the obtained hybrid beamforming design under this assumption in the second step requires updates to cancel the SI effectively, meeting the ideal SI cancellation constraint (8a). To cancel the SI, we first introduce the equivalent SI channel by fixing the obtained analog beamformer $F_{\mathrm{RFD}}$, the analog combiner $W_{\mathrm{RFU}}$, and the digital combiner $W_{\mathrm{BBU}}$ at the BS, which is defined as

(16) $$\begin{array}{c} H_{\mathrm{SIeq}}=W_{\mathrm{BBU}}^{H}W_{\mathrm{RFU}}^{H}H_{\mathrm{SI}}F_{\mathrm{RFD}}. \end{array}$$

Then, we leverage the null space projection (NSP), a widely used method in FD MIMO systems at the sub-6 GHz band [27,28] or the mmWave frequency [37,38,43,45], to cancel the SI. Let $C=N(H_{\mathrm{SIeq}})$ denote one set of orthogonal bases for the null space of the equivalent SI channel $H_{\mathrm{SIeq}}$. Then, the projection matrix onto this null space is

(19) $$\begin{array}{c} Q=C{\left(C^{H}C\right)}^{-1}C^{H}. \end{array}$$

We employ the NSP method by projecting the obtained digital beamformer $F_{\mathrm{BBD}}$ onto the null space of $H_{\mathrm{SIeq}}$ to null out the SI completely, which takes the form

(20) $$\begin{array}{c} F_{\mathrm{BBD}}=QF_{\mathrm{BBD}}. \end{array}$$

To satisfy the transmit power constraint (8c), we further normalize the obtained digital beamformer $F_{\mathrm{BBD}}$ by a factor of $\frac{\sqrt{P_{D}}}{∥F_{\mathrm{RFD}}F_{\mathrm{BBD}}{∥}_{F}}$. Thus far, the proposed three-step hybrid beamforming design to cancel the SI in the FD mmWave MIMO systems with DSs has been completed. We summarize the proposed hybrid beamforming design in Algorithm 1.
**Algorithm 1:** The Proposed Three-Step Hybrid Beamforming Design for SI Cancellation in the FD mmWave MIMO Systems with DSs1:**Input**$H_{U}$, $H_{D}$, $H_{\mathrm{SI}}$, $P_{U}$, $P_{D}$2:**Output:**$F_{\mathrm{RFU}}$, $F_{\mathrm{BBU}}$, $W_{\mathrm{RFU}}$, $W_{\mathrm{BBU}}$, $F_{\mathrm{RFD}}$, $F_{\mathrm{BBD}}$, $W_{\mathrm{RFD}}$, $W_{\mathrm{BBD}}$/∗ The first step ∗/3:Obtain the optimal fully digital solutions assuming no SI:$\left[U_{U},D_{U},V_{U}\right]=svd\left(H_{U}\right)$,    ${\tilde{F}}_{U}^{*}=V_{U}\left(:,1:N_{s}\right)T_{U}^{\frac{1}{2}}$,    ${\tilde{W}}_{U}^{*}=V_{U}\left(:,1:N_{s}\right)$;$\left[U_{D},D_{D},V_{D}\right]=svd\left(H_{D}\right)$,    ${\tilde{F}}_{D}^{*}=V_{D}\left(:,1:N_{s}\right)T_{D}^{\frac{1}{2}}$,    ${\tilde{W}}_{D}^{*}=V_{D}\left(:,1:N_{s}\right)$;/∗ The second step ∗/4:Initialize $F_{\mathrm{RFU}}$, $F_{\mathrm{RFD}}$, $W_{\mathrm{RFU}}$, and $W_{\mathrm{RFD}}$ randomly, satisfying constraints (6a–h) and (8d–g), $F_{\mathrm{BBU}}={\left(F_{\mathrm{RFU}}^{H}F_{\mathrm{RFU}}\right)}^{-1}F_{\mathrm{RFU}}^{H}{\tilde{F}}_{U}^{*}$, and $F_{\mathrm{BBD}}={\left(F_{\mathrm{RFD}}^{H}F_{\mathrm{RFD}}\right)}^{-1}F_{\mathrm{RFD}}^{H}{\tilde{F}}_{D}^{*}$.5:Obtain the final $F_{\mathrm{RFU}}$ and $F_{\mathrm{BBU}}$, $W_{\mathrm{RFU}}$ and $W_{\mathrm{BBU}}$, $F_{\mathrm{RFD}}$ and $F_{\mathrm{BBD}}$, and $W_{\mathrm{RFD}}$ and $W_{\mathrm{BBD}}$ according to the iterative hybrid beamforming design in Section 3.2;6:Power normalizations: $F_{\mathrm{BBU}}=\sqrt{P_{U}}\frac{F_{\mathrm{BBU}}}{∥F_{\mathrm{RFU}}F_{\mathrm{BBU}}{∥}_{F}}$,    $F_{\mathrm{BBD}}=\sqrt{P_{D}}\frac{F_{\mathrm{BBD}}}{∥F_{\mathrm{RFD}}F_{\mathrm{BBD}}{∥}_{F}}$;/∗ The third step ∗/7:Introduce the equivalent SI channel: $H_{\mathrm{SIeq}}=W_{\mathrm{BBU}}^{H}W_{\mathrm{RFU}}^{H}H_{\mathrm{SI}}F_{\mathrm{RFD}}$;8:Obtain the orthogonal bases C for the null space of $H_{\mathrm{SIeq}}$ and its projection matrix Q: $C=N(H_{\mathrm{SIeq}})$,    $Q=C{\left(C^{H}C\right)}^{-1}C^{H}$;9:NSP and power normalization: $F_{\mathrm{BBD}}=QF_{\mathrm{BBD}}$,    $F_{\mathrm{BBD}}=\sqrt{P_{D}}\frac{F_{\mathrm{BBD}}}{∥F_{\mathrm{RFD}}F_{\mathrm{BBD}}{∥}_{F}}$;10:**return** $F_{\mathrm{RFU}}$, $F_{\mathrm{BBU}}$, $W_{\mathrm{RFU}}$, $W_{\mathrm{BBU}}$, $F_{\mathrm{RFD}}$, $F_{\mathrm{BBD}}$, $W_{\mathrm{RFD}}$, $W_{\mathrm{BBD}}$

### 3.4. Computational Complexity Analysis

In this subsection, we analyze the computational complexity of the proposed three-step hybrid beamforming design for SI cancellation in FD mmWave MIMO systems with DCs, where the total required number of complex multiplications and additions is considered. For matrices $A\in C^{m\times n}$ and $B\in C^{n\times p}$, their product AB requires 2m(n−1)p complex float-point operations (FLOPs), including mnp complex multiplications and m(n−1)p complex additions. Considering the relationship among the number of antennas, RF chains, and data streams in the hybrid beamforming structures, the computational complexity of each step is provided as follows:

In the first step, the complexity of the SVD of UL and DL channels is much higher than that of the water-filling power allocation, yielding the complexity of this step as $O(M_{T}N_{R}^{2}+N_{T}^{2}M_{R})$. In the second step, the dynamic analog solutions $F_{\mathrm{RFU}}$, $F_{\mathrm{RFD}}$, $W_{\mathrm{RFU}}$, and $W_{\mathrm{RFD}}$ can be, respectively, updated with a worst-case complexity of $O(I_{1}M_{T}{\left(M_{\mathrm{RF}}^{t}\right)}^{2}+I_{1}I_{2}M_{T}{\left(M_{\mathrm{RF}}^{t0}\right)}^{2})$, $O(I_{1}N_{T}{\left(N_{\mathrm{RF}}^{t}\right)}^{2}+I_{1}I_{2}N_{T}{\left(N_{\mathrm{RF}}^{t0}\right)}^{2})$, $O(I_{1}N_{R}{\left(N_{\mathrm{RF}}^{r}\right)}^{2}+I_{1}I_{2}N_{R}{\left(N_{\mathrm{RF}}^{r0}\right)}^{2})$, and $O(I_{1}M_{R}{\left(M_{\mathrm{RF}}^{t}\right)}^{2}+I_{1}I_{2}M_{R}{\left(M_{\mathrm{RF}}^{r0}\right)}^{2})$, where $I_{1}$ and $I_{2}$ denote the number of the maximum iterations required in the second step and the maximum times of the extended Kuhn-Munkres algorithm used for the antenna reallocation, $M_{\mathrm{RF}}^{t0}$, $N_{\mathrm{RF}}^{r0}$, $N_{\mathrm{RF}}^{t0}$, and $M_{\mathrm{RF}}^{r0}$ are the number of RF chains connected to no antennas at MS1, the receiver of the BS, the transmitter of the BS, and MS2; the complexity of updating the digital solution $F_{\mathrm{BBU}}$, $F_{\mathrm{BBD}}$, $W_{\mathrm{BBU}}$, and $W_{\mathrm{BBD}}$ is $O(I_{1}M_{T}{\left(M_{\mathrm{RF}}^{t}\right)}^{2})$, $O(I_{1}N_{T}{\left(N_{\mathrm{RF}}^{t}\right)}^{2})$, $O(I_{1}N_{R}{\left(N_{\mathrm{RF}}^{r}\right)}^{2})$, and $O(I_{1}M_{R}{\left(M_{\mathrm{RF}}^{r}\right)}^{2})$, respectively. In the third step, the complexity of constructing the equivalent SI channel and employing NSP to cancel the SI is $O(N_{T}N_{R}N_{s}+{\left(N_{\mathrm{RF}}^{t}\right)}^{3})$. Let $M_{\mathrm{RF}}^{t}=M_{\mathrm{RF}}^{r}=M_{\mathrm{RF}}$, $N_{\mathrm{RF}}^{t}=N_{\mathrm{RF}}^{r}=N_{\mathrm{RF}}$, $M_{\mathrm{RF}}^{t0}=M_{\mathrm{RF}}^{r0}=M_{\mathrm{RF}}^{0}$, and $N_{\mathrm{RF}}^{t0}=N_{\mathrm{RF}}^{r0}=N_{\mathrm{RF}}^{0}$, and then the overall computational complexity of the proposed design is

(21) $$\begin{array}{cc} \; & O\left(M_{T}N_{R}^{2}+N_{T}^{2}M_{R}+I_{1}\left(\left(M_{T}+M_{R}\right)M_{\mathrm{RF}}^{2}+\left(N_{T}+N_{R}\right)N_{\mathrm{RF}}^{2}\right)\right.\\ \; & \;+\left.I_{1}I_{2}\left(\left(M_{T}+M_{R}\right){\left(M_{\mathrm{RF}}^{0}\right)}^{2}+\left(N_{T}+N_{R}\right){\left(N_{\mathrm{RF}}^{0}\right)}^{2}\right)\right)\\ \overset{\left(a\right)}{\approx } & O\left(M_{T}N_{R}^{2}+N_{T}^{2}M_{R}+I_{1}\left(N_{T}+N_{R}\right)N_{\mathrm{RF}}^{2}+I_{1}I_{2}\left(N_{T}+N_{R}\right){\left(N_{\mathrm{RF}}^{0}\right)}^{2}\right), \end{array}$$

where (a) is the result of assuming the number of antennas, RF chains, and the RF chains connected to no antennas at the BS is, respectively, much larger than the corresponding number at MSs.

We further compare the computational complexity of our proposed design with **BFC_FCS** (the proposed hybrid beamforming cancellation method for FD mmWave MIMO systems with FCSs in [37]) and **SIC_FS** (the SI cancellation-based hybrid beamforming design for FD mmWave MIMO systems with FSs in [45]). For a fair comparison, under the same assumption as in (21), the complexity of the BFC_FCS method is $O(M_{T}N_{R}^{2}+N_{T}^{2}M_{R}+(\left(N_{T}+N_{R}\right)+2|D|)|D|N_{\mathrm{RF}}N_{s}+\left(N_{T}+N_{R}\right)N_{\mathrm{RF}}^{3})$, where D denotes the codebook set used for analog beamforming and combining; the complexity of the SIC_FS method is $O(M_{T}N_{R}^{2}+N_{T}^{2}M_{R}+I_{3}{\left(N_{\mathrm{RF}}N_{s}+1\right)}^{4.5}\log\left(1/\varepsilon \right))$, where $I_{3}$ denotes the maximum iterations of the SDR-AltMin method in [12] used to obtain the hybrid beamforming design, and ε is the solution accuracy of the semidefinite relaxation (SDR) problem.

The computational complexities of the three methods are listed in Table 1. From the expressions of the complexity in this table, we obtain that the BFC_FCS method and our proposed design have comparable complexity. This is because the former has a higher-order term $O(\left(N_{T}+N_{R}\right)N_{\mathrm{RF}}^{3})$ and |D|; the number of elements in the codebook set is usually larger than the number of RF chains, while the latter requires iterations. Considering the hybrid beamforming design for DSs is more challenging than for FCSs, our proposed design has its own advantages. As the SIC_FS method has a higher-order term $O({\left(N_{\mathrm{RF}}N_{s}+1\right)}^{4.5}\log\left(1/\varepsilon \right))$ in its complexity and requires calling the CVX toolbox [50] to solve the SDR problem, it usually has a longer running time than our proposed method.

## 4. Numerical Results

In this section, we present the numerical results to evaluate the performance of the proposed hybrid beamforming design for the FD mmWave MIMO systems with DSs. For comparison, we consider the following classical methods as benchmarks: **BFC_FCS** [37], **SIC_FS** [45], **Ideal FD** (the fully digital beamforming for FD mmWave MIMO systems assuming no SI), and **Ideal HD** (the fully digital beamforming using all antennas at BS for HD mmWave communications).

In the simulations, we assume the carrier frequency is $f_{c}=28\;\mathrm{GHz}$. The UL and DL channels are set as $N_{c}=6$ clusters with $N_{l}=15$ propagation paths per cluster. The AoAs/AoDs of the propagation paths in the same cluster follow the Laplacian distribution with the mean angle uniformly distributed in [0,2π] and the angular spread of 10. We consider the uniform linear arrays (ULAs) with the antenna spacing $d=\frac{\lambda }{2}$. Thus, the normalized receive and transmit antenna array response vectors in (3) are expressed as

(22a) $$a_{r}\left(\theta_{cl}^{r}\right)=\frac{1}{\sqrt{N_{r}}}{\left[1,e^{j\pi \sin\left(\theta_{cl}^{r}\right)},e^{j2\pi \sin\left(\theta_{cl}^{r}\right)},\ldots ,e^{j\left(N_{r}-1\right)\pi \sin\left(\theta_{cl}^{r}\right)}\right]}^{T},$$

(22b) $$a_{t}\left(\theta_{cl}^{t}\right)=\frac{1}{\sqrt{N_{t}}}{\left[1,e^{j\pi \sin\left(\theta_{cl}^{t}\right)},e^{j2\pi \sin\left(\theta_{cl}^{t}\right)},\ldots ,e^{j\left(N_{t}-1\right)\pi \sin\left(\theta_{cl}^{t}\right)}\right]}^{T}.$$

The distance between the BS and MS1 (MS2) is assumed to be $d_{n}=40\;m$, and thus, according to [51], the path loss $\gamma =61.4+20\log_{10}\left(d_{n}\right)+\xi \;\left[\mathrm{dB}\right]$, where $\xi ∼N(0,5.8^{2})$. For the SI channel at BS, we set the Rician factor as κ=30dB and the distance and angle between the transmit and receive antenna arrays as 20λ and $\frac{\pi }{6}$, respectively. Unless mentioned otherwise, the BS is equipped with $N_{T}=32$ transmit antennas, $N_{R}=32$ receive antennas, and $N_{\mathrm{RF}}^{t}=N_{\mathrm{RF}}^{r}=4$ RF chains. MS1 and MS2 are, respectively, equipped with $M_{T}=16$ transmit antennas and $M_{R}=16$ receive antennas, and the number of their RF chains is $M_{\mathrm{RF}}^{t}=M_{\mathrm{RF}}^{r}=4$. We set the number of data streams $N_{s}=3$. The maximum transmit power at MS1 and the BS is, respectively, set to $P_{U}=23\;\mathrm{dBm}$ and $P_{D}=30\;\mathrm{dBm}$. The signal-to-noise ratio (SNR) is defined by $\mathrm{SNR}=10\log_{10}\left(\frac{P_{rx}}{\sigma^{2}}\right)\;\mathrm{dB}$, where $P_{rx}$ denotes the received power and $\sigma^{2}$ is the noise power. For simplicity, we assume the SNR at BS is equal to that at MS2. Each simulation result is obtained by averaging 500 random channel realizations. The above default simulation parameters are listed in Table 2.

### 4.1. Spectral Efficiency

**Figure 2:**
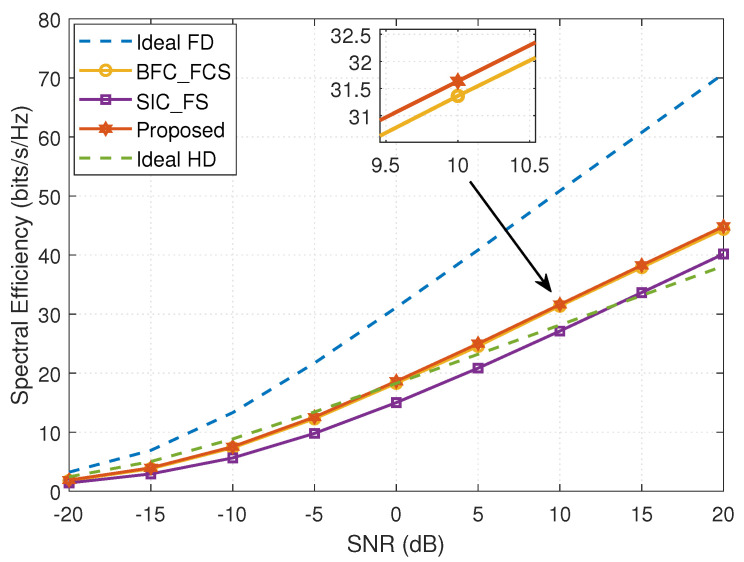
The spectral efficiency achieved by different methods versus SNR ($M_{T}=16$, $N_{T}=32$, $N_{R}=32$, $M_{R}=16$, $N_{\mathrm{RF}}^{t}=N_{\mathrm{RF}}^{t}=4$, $M_{\mathrm{RF}}^{t}=M_{\mathrm{RF}}^{t}=4$, $N_{s}=3$, $P_{U}=23\;\mathrm{dBm}$, and $P_{D}=30\;\mathrm{dBm}$).

Figure 2 illustrates the SE achieved by different methods versus SNR. We observe that the FD mmWave MIMO system with DSs using the proposed design can achieve an SE close to even a little better than the system with FCSs using the BFC_FCS method. The main reasons are two-fold: on the one hand, as the dimension of the equivalent channel $H_{\mathrm{SIeq}}$ in this simulation is 3×4, according to the rank-nullity theorem, the dimension of its null space is very low, which seriously limits the SI cancellation of the FCSs and DSs; on the other hand, to obtain the hybrid beamforming design from the optimal fully digital solutions obtained assuming no SI, the BFC_FCS method, exploiting the spatially sparse precoding in [11], can only update the analog beamformers or combiners column-by-column, but our proposed design can update every nonzero element in the analog solutions independently. Thus, our proposed design achieves slightly better SI cancellation in the FD mmWave MIMO system with DSs, leading to a better SE. We can also see that the DSs achieve better SE than FSs in the FD mmWave MIMO system, mainly due to the higher beamforming gains provided by DSs. When SNR>0dB, the FD mmWave system with FCSs or DSs can achieve better SE than Ideal HD; when SNR>12dB, the system with FSs is superior to Ideal HD. When SNR=20dB, the FD mmWave MIMO system with DSs using the proposed design outperforms Ideal HD by approximately 17.56%.

**Figure 3:**
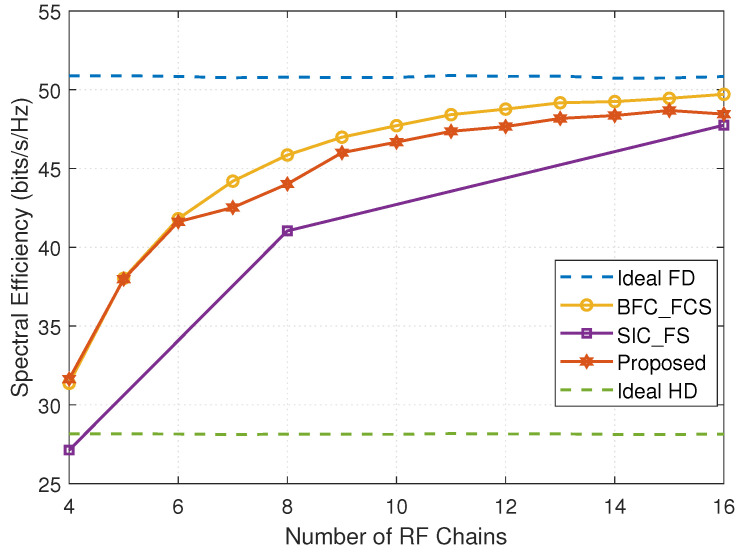
The spectral efficiency versus the number of RF chains ($M_{T}=16$, $N_{T}=32$, $N_{R}=32$, $M_{R}=16$, $N_{s}=3$, $P_{U}=23\;\mathrm{dBm}$, $P_{D}=30\;\mathrm{dBm}$, and SNR=10dB).

Figure 3 plots the SE versus the number of RF chains, where $M_{\mathrm{RF}}^{t}=N_{\mathrm{RF}}^{t}=N_{\mathrm{RF}}^{r}=M_{\mathrm{RF}}^{r}$. It is observed that with the increase in the number of RF chains, the achievable SE improves. The main reason is that when increasing the number of RF chains and fixing the number of data streams, the dimension of the null space of the equivalent channel $H_{\mathrm{SIeq}}$ increases, leading to better SI cancellation. When the number of RF chains is fewer than 6, the SE of the FD mmWave MIMO system with DSs is close to that of the system with FCSs, the probable reasons for which are similar to those in Figure 2; when the number of RF chains is larger than 6, the system with FCSs achieves better SE than that with DSs, for the former structure enjoys full beamforming gains. The SE achieved by the FD mmWave system with DSs using the proposed design is much higher than that of the system with FSs and Ideal HD. However, when the number of RF chains is small, the SE of the FD mmWave MIMO system with FSs is inferior to Ideal HD.

**Figure 4:**
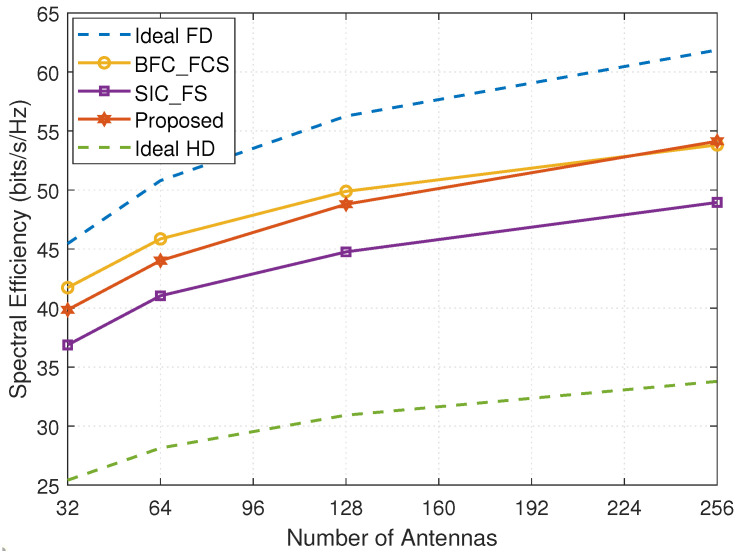
The spectral efficiency versus the number of antennas at the BS ($M_{T}=16$, $M_{R}=16$, $N_{s}=3$, $N_{\mathrm{RF}}^{t}=N_{\mathrm{RF}}^{t}=8$, $M_{\mathrm{RF}}^{t}=M_{\mathrm{RF}}^{t}=8$, $P_{U}=23\;\mathrm{dBm}$, $P_{D}=30\;\mathrm{dBm}$, and SNR=10dB).

Figure 4 presents the achievable SE versus the number of antennas at the BS. We assume the BS has the same number of transmit and receive antennas, i.e., half of the antennas are dedicated to transmission and the other half to reception. We see that the achievable SE increases with the number of antennas, since more antennas can provide better antenna diversity and higher beamforming gains. With the increase in the number of antennas at the BS, the SE gap between the system with FCSs and DSs decreases. Especially when the number of antennas is greater than approximately 225, the FD mmWave MIMO system with DSs using the proposed design achieves better SE than the system with FCSs resorting to the BFC_FCS method. The probable reason is that when the number of antennas is large, updating every nonzero element of the analog solutions for DSs independently and separately leads to better beamforming gains and SI cancellation than updating the analog solutions for FCSs column-by-column via the spatially sparse precoding [11]. The FD mmWave system with DSs achieves much better SE than the system with FSs and Ideal HD. When the number of antennas at the BS is 256, the SE of the FD mmWave MIMO systems with DSs is about 69.27% higher than that of the Ideal HD.

### 4.2. Energy Efficiency

Taking the power consumption into consideration, EE is another important performance of mmWave communications. In this subsection, we evaluate the EE of FD mmWave MIMO systems with different hybrid beamforming structures. The EE is defined as the ratio of the overall system SE and the total power consumption $P_{total}$, which is given by

(23) $$\begin{array}{c} E=\frac{R_{U}+R_{D}}{P_{total}}. \end{array}$$

To evaluate the EE accurately, the total power consumption $P_{total}$ includes not only the transmit power $P_{U}$ and $P_{D}$ but also the power of the hardware circuits, which are the baseband processor power $P_{\mathrm{BB}}$, the digital-to-analog converter power $P_{\mathrm{DAC}}$, the ADC power $P_{\mathrm{ADC}}$, the RF chain power $P_{\mathrm{RF}}$, the phase shifter power $P_{\mathrm{PS}}$, the power amplifier power $P_{\mathrm{PA}}$, the low-noise amplifier power $P_{\mathrm{LNA}}$, and the switch power $P_{\mathrm{SW}}$. The specific expressions of $P_{total}$ for the FD mmWave MIMO system with different structures are listed in Table 3, where $P_{C}=P_{U}+P_{D}+4P_{\mathrm{BB}}+\left(M_{\mathrm{RF}}^{t}+N_{\mathrm{RF}}^{t}\right)P_{\mathrm{DAC}}+\left(M_{\mathrm{RF}}^{r}+N_{\mathrm{RF}}^{r}\right)P_{\mathrm{ADC}}+\left(M_{\mathrm{RF}}^{t}+N_{\mathrm{RF}}^{t}+M_{\mathrm{RF}}^{r}+N_{\mathrm{RF}}^{r}\right)P_{\mathrm{RF}}+\left(M_{T}+N_{T}\right)P_{\mathrm{PA}}+\left(M_{R}+N_{R}\right)P_{\mathrm{LNA}}$ denotes the common dissipated powers in the systems with different hybrid beamforming structures. In the simulations, we set $P_{\mathrm{BB}}=200\;\mathrm{mW}$, $P_{\mathrm{DAC}}=P_{\mathrm{ADC}}=200\;\mathrm{mW}$, $P_{\mathrm{RF}}=40\;\mathrm{mW}$, $P_{\mathrm{PS}}=30\;\mathrm{mW}$, $P_{\mathrm{PA}}=150\;\mathrm{mW}$, $P_{\mathrm{LNA}}=20\;\mathrm{mW}$, and $P_{\mathrm{SW}}=5\;\mathrm{mW}$ [52].

**Figure 5:**
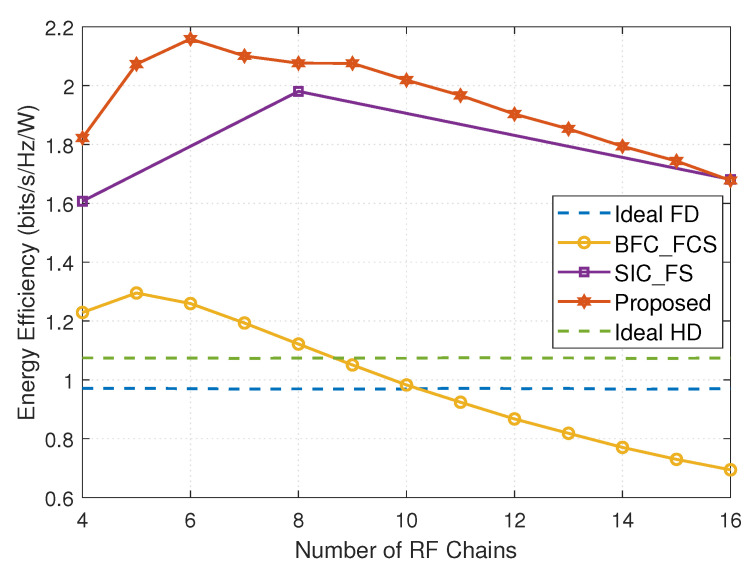
The energy efficiency versus the number of RF chains ($M_{T}=16$, $N_{T}=32$, $N_{R}=32$, $M_{R}=16$, $N_{s}=3$, $P_{U}=23\;\mathrm{dBm}$, $P_{D}=30\;\mathrm{dBm}$, and SNR=10dB).

Figure 5 shows the EE of FD mmWave MIMO systems with different hybrid beamforming structures versus the number of RF chains, where $M_{\mathrm{RF}}^{t}=N_{\mathrm{RF}}^{t}=N_{\mathrm{RF}}^{r}=M_{\mathrm{RF}}^{r}$. We observe that the system with DSs achieves better EE than those with FSs and FCSs, as well as Ideal FD and Ideal HD. With the increase in the number of RF chains, the EE of the system with DSs first increases and then decreases, reaching its maximum at $M_{\mathrm{RF}}^{t}=N_{\mathrm{RF}}^{t}=N_{\mathrm{RF}}^{r}=M_{\mathrm{RF}}^{r}=6$. The main reason is that when the number of RF chains is larger than 6, SE increases slowly, but the power consumption increases constantly, and thus, EE decreases. For the FD mmWave systems with FSs and FCSs, their EE has similar changes to the system with DSs, and their maximum EE is obtained at the number of RF chains equal to 5 and 8, respectively. In addition, the EE achieved by the FD mmWave system with FSs is higher than that with FCSs, mainly due to the large number of phase shifters required in the FCSs. The EE gap between the systems with DSs and FSs first increases and then decreases. When the number of RF chains is 6, the EE gap is the largest; when the number of RF chains is 16, the systems with DSs and FSs achieve very close EE. The number of RF chains in the system with FCSs to enjoy better EE than Ideal HD and Ideal FD is fewer than 8 and 10, respectively. Therefore, the FD mmWave MIMO system with DSs is a preferable solution for future green communications with critical requirements on high EE.

## 5. Conclusions

In this paper, we introduced the DSs to the FD mmWave MIMO system and proposed an effective three-step hybrid beamforming design for SI cancellation in the considered system, which can guarantee that each RF chain is connected to at least one antenna and effectively cancels the SI. The computational complexity of the proposed design was provided. The numerical results demonstrated that the FD mmWave MIMO system with DSs using the proposed design can achieve better SE than the system with FSs and much better EE than Ideal FD, Ideal HD, and that with FCSs or FSs. The main reason for the superiority of the FD mmWave MIMO system with DSs using the proposed design is that the DSs require that each antenna is only connected to one RF chain and each RF chain is connected to at least one antenna. Thus, fewer phase shifters are used compared to FCSs, and the corresponding analog beamformer/combiner, each row of which has only one nonzero element and each column of which has at least one nonzero element, is not a static block diagonal matrix as for FSs. Hence, the DSs provide dynamic connections between antennas and RF chains, leading to better SI cancellation and thus improving SE and EE.

In future works, we will further investigate the hybrid beamforming design for the FD mmWave MIMO systems with DSs considering the limited dynamic ranges of ADCs, imperfect CSI, and the inter-user interference. Furthermore, to reduce the high energy cost, the hybrid beamforming design for the FD mmWave MIMO systems with DSs using renewable energy via energy harvesting also deserves further investigation.

## Figures and Tables

**Table 1:** Computational Complexity of Different Methods.

Methods	Computational Complexity
BFC_FCS	$O(M_{T}N_{R}^{2}+N_{T}^{2}M_{R}+(\left(N_{T}+N_{R}\right)+2|D|)|D|N_{\mathrm{RF}}N_{s}+\left(N_{T}+N_{R}\right)N_{\mathrm{RF}}^{3})$
SIC_FS	$O(M_{T}N_{R}^{2}+N_{T}^{2}M_{R}+I_{3}{\left(N_{\mathrm{RF}}N_{s}+1\right)}^{4.5}log\left(1/\varepsilon \right))$
Proposed	$O(M_{T}N_{R}^{2}+N_{T}^{2}M_{R}+I_{1}\left(N_{T}+N_{R}\right)N_{\mathrm{RF}}^{2}+I_{1}I_{2}\left(N_{T}+N_{R}\right){\left(N_{\mathrm{RF}}^{0}\right)}^{2})$

**Table 2:** Simulation Parameters.

Parameters	Values
Carrier frequency and wavelength $[f_{c}$, λ]	[28GHz,0.0107m]
Number of clusters and rays $[N_{c}$, $N_{l}]$	[6, 15]
AoAs $\left[a_{r}\left(\theta_{cl}^{r}\right)\right]$ / AoDs $\left[a_{t}\left(\theta_{cl}^{t}\right)\right]$	U[0,2π]
Angular spread of rays in the same cluster	$10^{}$
Distance between BS and MS1 (MS2) $\left[d_{n}\right]$	40m
Path loss [γ]	RefertoTableIin [51]
Distance and angles between antenna arrays at FD BS $[d_{0}$, Θ]	$\left[20\lambda ,\frac{\pi }{6}\right]$
Rician factor [κ]	[30dB]
Number of antennas $[M_{T}$, $N_{T}$, $N_{R}$, $M_{R}]$	[16,32,32,16]
Number of RF chains $[M_{\mathrm{RF}}^{t}$, $N_{\mathrm{RF}}^{t}$, $N_{\mathrm{RF}}^{r}$, $M_{\mathrm{RF}}^{r}]$	[4,4,4,4]
Number of data streams $\left[N_{s}\right]$	3
Maximum transmit powers $[P_{U}$, $P_{D}]$	[23dBm,30dBm]
Number of Monte Carlo simulations	500

**Table 3:** Total Power Consumption for the FD mmWave MIMO Systems with Different Hybrid Beamforming Structures.

mmWave Structures	Total Power Consumption $P_{\mathrm{total}}$
FCSs	$P_{C}+\left(M_{\mathrm{RF}}^{t}M_{T}+N_{\mathrm{RF}}^{t}N_{T}+M_{\mathrm{RF}}^{r}M_{R}+N_{\mathrm{RF}}^{r}N_{R}\right)P_{\mathrm{PS}}$
FSs	$P_{C}+\left(M_{T}+N_{T}+M_{R}+N_{R}\right)P_{\mathrm{PS}}$
DSs	$P_{C}+\left(M_{T}+N_{T}+M_{R}+N_{R}\right)P_{\mathrm{PS}}+\left(\left(M_{T}+N_{T}+M_{R}+N_{R}\right)\right)P_{\mathrm{SW}}$

## Data Availability

Not applicable.
